# Risk–benefit ratio of percutaneous kyphoplasty and percutaneous vertebroplasty in patients with newly diagnosed multiple myeloma with vertebral fracture: a single-center retrospective study

**DOI:** 10.1007/s00277-023-05202-9

**Published:** 2023-03-30

**Authors:** Qiu-Qing Xiang, Bin Chu, Min-Qiu Lu, Lei Shi, Shan Gao, Yu-Tong Wang, Li-Juan Fang, Yue-Hua Ding, Xin Zhao, Yuan Chen, Meng-Zhen Wang, Wei-Kai Hu, Li-Fang Wang, Kai Sun, Li Bao

**Affiliations:** 1 Department of Hematology, Beijing Jishuitan Hospital, Beijing, China; 2 Department of Epidemiology and Statistics, Beijing Jishuitan Hospital, Beijing, China

**Keywords:** Multiple myeloma, Percutaneous vertebroplasty, Percutaneous kyphoplasty, Overall survival

## Abstract

The indications for percutaneous kyphoplasty (PKP) and percutaneous vertebroplasty (PVP) are painful vertebral compression fractures. Our study is to assess the risk–benefit ratio of PKP/PVP surgery in the patients with newly diagnosed multiple myeloma (NDMM) without receiving antimyeloma therapy. The clinical data of 426 consecutive patients with NDMM admitted to our center from February 2012 to April 2022 were retrospectively analyzed. The baseline data, postoperative pain relief, the proportion of recurrent vertebral fractures, and survival time were compared between the PKP/PVP surgical group and the nonsurgical group in the NDMM patients. Of the 426 patients with NDMM, 206 patients had vertebral fractures (206/426, 48.4%). Of these, 32 (32/206, 15.5%) underwent PKP/PVP surgery for misdiagnosis of simple osteoporosis prior to diagnosis of MM (surgical group), and the other 174 (174/206, 84.5%) did not undergo surgical treatment prior to definitive diagnosis of MM (non-surgical group). The median age of patients in the surgical and nonsurgical groups was 66 and 62 years, respectively (*p* = 0.01). The proportion of patients with advanced ISS and RISS stages was higher in the surgical group (ISS stage II + III 96.9% vs. 71.8%, *p* = 0.03; RISS stage III 96.9% vs. 71%, *p* = 0.01). Postoperatively, 10 patients (31.3%) never experienced pain relief and 20 patients (62.5%) experienced short-term pain relief with a median duration of relief of 2.6 months (0.2–24.1 months). Postoperative fractures of vertebrae other than the surgical site occurred in 24 patients (75%) in the surgical group, with a median time of 4.4 months postoperatively (0.4–86.8 months). Vertebral fractures other than the fracture site at the first visit occurred in 5 patients (2.9%) in the nonoperative group at the time of diagnosis of MM, with a median time of 11.9 months after the first visit (3.5–12.6 months). The incidence of secondary fractures was significantly higher in the surgical group than in the nonsurgical group (75% vs. 2.9%, *p* = 0.001). The time interval between the first visit and definitive diagnosis of MM was longer in the surgical group than in the nonsurgical group (6.1 months vs. 1.6 months, *p* = 0.01). At a median follow-up of 32 months (0.3–123 months), median overall survival (OS) was significantly shorter in the surgical group than in the nonsurgical group (48.2 months vs. 66 months, *p* = 0.04). Application of PKP/PVP surgery for pain relief in NDMM patients without antimyeloma therapy has a limited effect and a high risk of new vertebral fractures after surgery. Therefore, patients with NDMM may need to have their disease controlled with antimyeloma therapy prior to any consideration for PKP/PVP surgery.

## Introduction

Multiple myeloma (MM) is a hematologic malignancy in which abnormal plasma cells proliferate clonally in the bone marrow and is the second most common tumor of the hematologic system, with a median age of onset of 70 years [1].

Myeloma bone disease (MBD) is a bone destructive lesion caused by MM cells, which manifests clinically as osteoporosis, hypercalcemia, osteolytic destruction, and pathological fractures. In severe cases, it can cause severe bone pain and spinal cord compression. Several consensus guidelines indicate that MBD should be treated mainly with bone-targeting drugs such as bisphosphonates or denosumab [2–5]. Zoledronic acid is the bone targeting drug of choice for MM patients, and denosumab is preferred for patients with renal insufficiency [2]. Antineoplastic drugs for myeloma such as proteasome inhibitors, immunomodulators, and monoclonal antibodies may also improve osteolytic lesions [6]. The Bone Working Group of the International Myeloma Working Group also found cement augmentation to be effective for painful vertebral compression fractures [2].

Percutaneous vertebroplasty (PVP) involves the percutaneous injection of bone cement into the fractured vertebral body to stabilize the vertebral body and destroy nerve endings for pain relief[7]. Percutaneous kyphoplasty (PKP) was developed from PVP. PKP uses a balloon to create a cavity in the vertebral body to increases safety and offers the option of restoring vertebral body height [8]. The indications for percutaneous kyphoplasty (PKP) and percutaneous vertebroplasty (PVP) are painful vertebral compression fractures. PKP/PVP surgery can quickly relieve pain and stabilize the vertebral body, and several foreign studies have shown that PKP/PVP is safe and effective in MM patients with vertebral pain [9–16]. However, these studies were reported from orthopedic surgeons and lacked information on antimyeloma treatment and long-term survival follow-up. A consensus statement from the International Myeloma Working Group (IMWG) has recommended the use of PKP/PVP in the setting of myeloma bone disease [17]. Myeloma is a frequently misdiagnosed hematologic malignancy, but the risk–benefit ratio of performing PKP/PVP prior to diagnosis in myeloma patients remains relatively unknown. This study analyzed the clinical benefit and risk of PKP/PVP surgery for patients with NDMM without antimyeloma therapy in a retrospective study to provide a reference for the rational use of PKP/PVP in MM patients.

## Materials and methods

### General information

This study is a single-center retrospective observational study. We analyzed 426 patients with NDMM who were seen in our department from February 2012 to April 2022 and had complete follow-up data. The diagnostic criteria were based on the 2014 IMWG diagnostic criteria [18]. Vertebral fractures were diagnosed according to the imaging manifestations of X-ray, CT, MRI, and PET-CT. Whenever possible, our patients will undergo spinal MRI STIR to identify skeletal lesions. PET-CT is performed in patients with extramedullary masses. X-rays or CT are used for localized lesions in the head or extremities. The differences in clinical information such as age, gender, time interval between first visit and diagnosis of MM, clinical stage (including DS stage [19], ISS stage [20], and RISS stage [21]) and overall survival (OS), and surgical complications were compared between the PKP/PVP surgical and nonsurgical groups. All patients signed an informed consent form at the time of treatment, and this study complied with the requirements of our institutional ethics committee (JST202109-02).

### Statistical methods

All data were analyzed by SPSS 19.0 statistical software. Nonnormally distributed data were expressed as median (range). Comparisons between groups were performed by nonparametric *u*-test. Univariate and multifactorial analyses affecting patient survival were performed by the Cox model. Survival curves were plotted by the Kaplan–Meier method, and survival differences between two groups were analyzed by log-rank test, and statistical differences were considered at *p* < 0.05. Definition of OS: From diagnosis of multiple myeloma to death or follow-up endpoint.

## Results

### Patient characteristics

Of the 426 patients with NDMM, 206 patients had vertebral fractures (206/426, 48.4%). Of these, 32 (32/206, 15.5%) underwent PKP/PVP surgery for misdiagnosis of simple osteoporosis prior to diagnosis of MM (surgical group), and the other 174 (174/206, 84.5%) did not undergo surgical treatment prior to definitive diagnosis of MM (nonsurgical group).

Baseline information for both groups is shown in Table 1: compared to the nonsurgical group, the surgical group had a higher median age (66 vs. 62 years, *p* = 0.01) and a higher proportion of patients with advanced ISS and advanced RISS (stage II + III ISS 96.9% vs. 71.8%, *p* = 0.03; stage III RISS 96.9% vs. 71%, *p* = 0.01). Vertebral fracture sites and surgical sites in 32 patients in the surgical group are shown in Table 2.

**Table 1:** Basic data of 206 newly diagnosed MM patients with vertebral body fracture

	Surgery group (*N* = 32)	Nonsurgical group (*N* = 174)	*p* value
Age	66 (51–83)	62 (30–78)	0.001
Gender			0.471
Male	16 (50%)	99 (56%)	
Female	16 (50%)	75 (43%)	
Anemia	22 (68.8%)	124 (71.3%)	0.774
Renal insufficiency	5 (15.6%)	25 (14.4%)	0.853
Hypercalcemia	5 (15.6%)	30 (17.2%)	0.823
ISS staging			0.057
I	1 (3.1%)	49 (28.2%)	
II	15 (46.9%)	51 (29.3%)	
III	16 (50%)	74 (42.5%)	
RISS staging			0.085
I	1 (3%)	36 (20.7%)	
II	26 (81.3%)	97 (55.7%)	
III	5 (15.6%)	25 (14.4%)	
Fracture location			0.203
Cervical vertebra	0	5 (2%)	
Thoracic vertebra	16 (50%)	55 (31.6%)	
Lumbar vertebra	7 (21.9%)	58 (33.3%)	
Thoracic combined with lumbar vertebra	9 (28.1%)	56 (32.2%)	
Time from initial diagnosis to diagnosis of MM (months)	6.1 (0.5 ~ 88)	1.6 (0.1 ~ 36.9)	0.001
O S (month)	48.2	66	0.04

*ISS* International Staging System, *RISS* Revised International Staging System, *OS* overall survival

**Table 2:** Vertebral fracture sites and surgical sites in 32 patients in the surgical group

Case#	Fracture site at onset	First surgical sites	Site of new vertebral fractures after first surgery	Second surgical sites	Site of new vertebral fractures after second surgery
1	T11	T11	–	–	–
2	T12 L2	T12 L2	T7-11 L3-5	–	–
3	T12	T12	T11 L1	–	–
4	T12 L1	T12 L1	T8-11 L2 L4 L5	–	–
5	T12 L1 L4	L1 L4	T11 L3	–	–
6	T11	T11	L1 L2	–	–
7	T7-9 T11-12 L1-3	T7 T9 T11	T2-6 L4	–	–
8	L1	L1	T7	T7	T11
9	L1	L1	T12	–	–
10	T12	T12	–	–	–
11	L1-2	L1	–	–	–
12	T7-8	T8	T12	T12	–
13	T5	T5	–	–	–
14	T10-11	T10-11	L1	L1	–
15	T12 L1	L1	–	T12	
16	T6 T8	T6 T8	T12	T12	T3 T11
17	T8	T8	L1-2	L1-2	T12 L3
18	T12	T12	–	–	–
19	T4 T7 T12 L1	T4 T7 T12 L1	–	–	–
20	T6 T8	T6 T8	T7	T7	–
21	T8-10 L1-5	L1 L2 L3 L5	T3 T6 T7	–	–
22	T9	T9	T7 T12	T7 T12	T11 L1
23	T12	T12	T10	–	–
24	L1	L1	T2 T8-9 T12 L2	–	–
25	L1-2	L1-2	T6	–	–
26	T12 L2	T12	T7-8 T11	–	–
27	T8	T8	T5 T7	–	–
28	T10 T12 L1 L4	T10 T12	–	–	–
29	T8 T10 T12 L2	T12 L2	T7 T9 T11 L1	–	–
30	L1	L1	T7-8	–	–
31	L1 L3-4	L1 L3-4	T6-12		
32	T12	T12	T11	T11	T10

*T* thoracic vertebra, *L* lumbar vertebra, *PVP* percutaneous vertebroplasty, *PKP* percutaneous kyphoplasty

### Postoperative pain relief rate in the surgical group

**Fig. 1:**
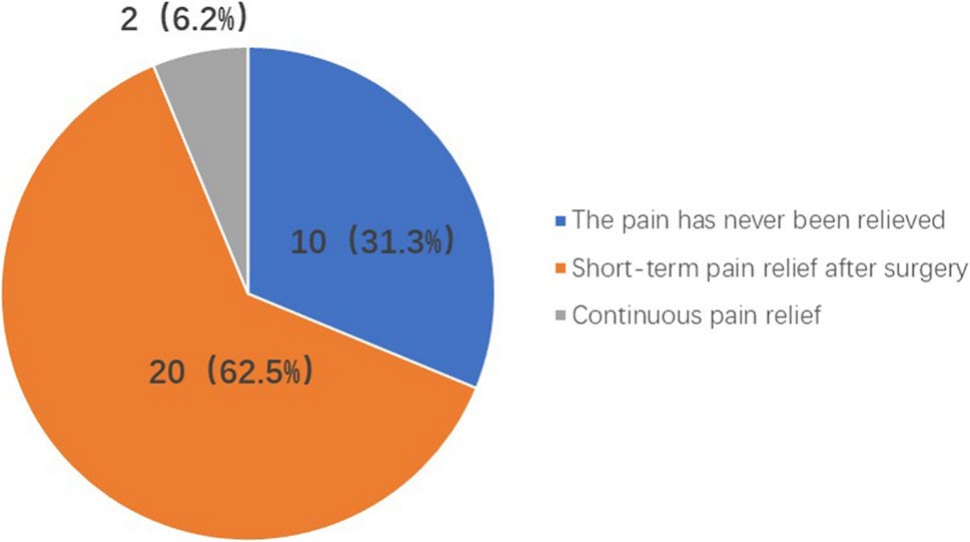
Postoperative pain relief of percutaneous vertebroplasty and percutaneous kyphoplasty. 10 of 32 patients (31.3%) never experienced pain relief after surgery, 20 (62.5%) experienced pain relief for a short period of time, and a few patients (2, 6.2%) achieved sustained pain relief

10 of 32 patients (31.3%) never experienced pain relief after surgery, 20 (62.5%) experienced pain relief for a short period of time with a median time to relief of 2.6 months (0.2–24.1), and a few patients (2, 6.2%) achieved sustained pain relief. See Fig. 1 for details.

### The incidence of new fractures

The differences in new fractures between the surgical and nonoperative groups prior to the diagnosis of MM are shown in Table 3. 24 of 32 patients (75%) in the surgical group developed new vertebral fractures postoperatively, with a median time to postoperative resolution of 4.4 months (0.4–86.8 months). Five of 174 patients (2.9%) in the nonoperative group had a vertebral fracture other than the site of the fracture at the time of the first visit at the time of diagnosis of MM, with a median time since the first visit of 11.9 months (3.5–12.6). The incidence of new fractures was significantly higher in the surgical group compared with the nonoperative group (75% vs. 2.9%, *p* = 0.001).

**Table 3:** Recurrence of fractures in the surgical and nonsurgical groups

	Surgery group (*N* = 32)	Postoperative time (months)	Nonsurgical group (*N* = 174)	Occurrence time after first visit (months)
Recurring fracture	24 (75%)	4.4 (0.4 ~ 86.8)	5 (2.9%)	11.9 (3.5–12.6)
Adjacent vertebral fractures	5 (20.8%)	3 (0.5 ~ 4.6)	1 (20%)	3.9
Nonadjacent vertebral fractures	8 (33.3%)	12.3 (1.8 ~ 86.8)	4 (80%)	11.9 (3.5–12.6)
Adjacent and nonadjacent vertebral fractures	11 (45.9%)	.92 (0.4 ~ 6.1)	0	–

### Median time to diagnosis of MM

In the surgical group, the diagnosis of MM was made after surgery in all 32 patients. The time interval from first visit to definitive diagnosis of MM was longer in the surgical group compared to the nonsurgical group (6.1 months vs. 1.6 months, *p* = 0.01).

### Higher risk–benefit ratio in 2 patients with PKP/PVP

Two patients (6.2%) in the surgical group developed paravertebral masses and paraplegia, respectively, after surgery. Detailed information is shown below.

#### Case 1

**Fig. 2:**
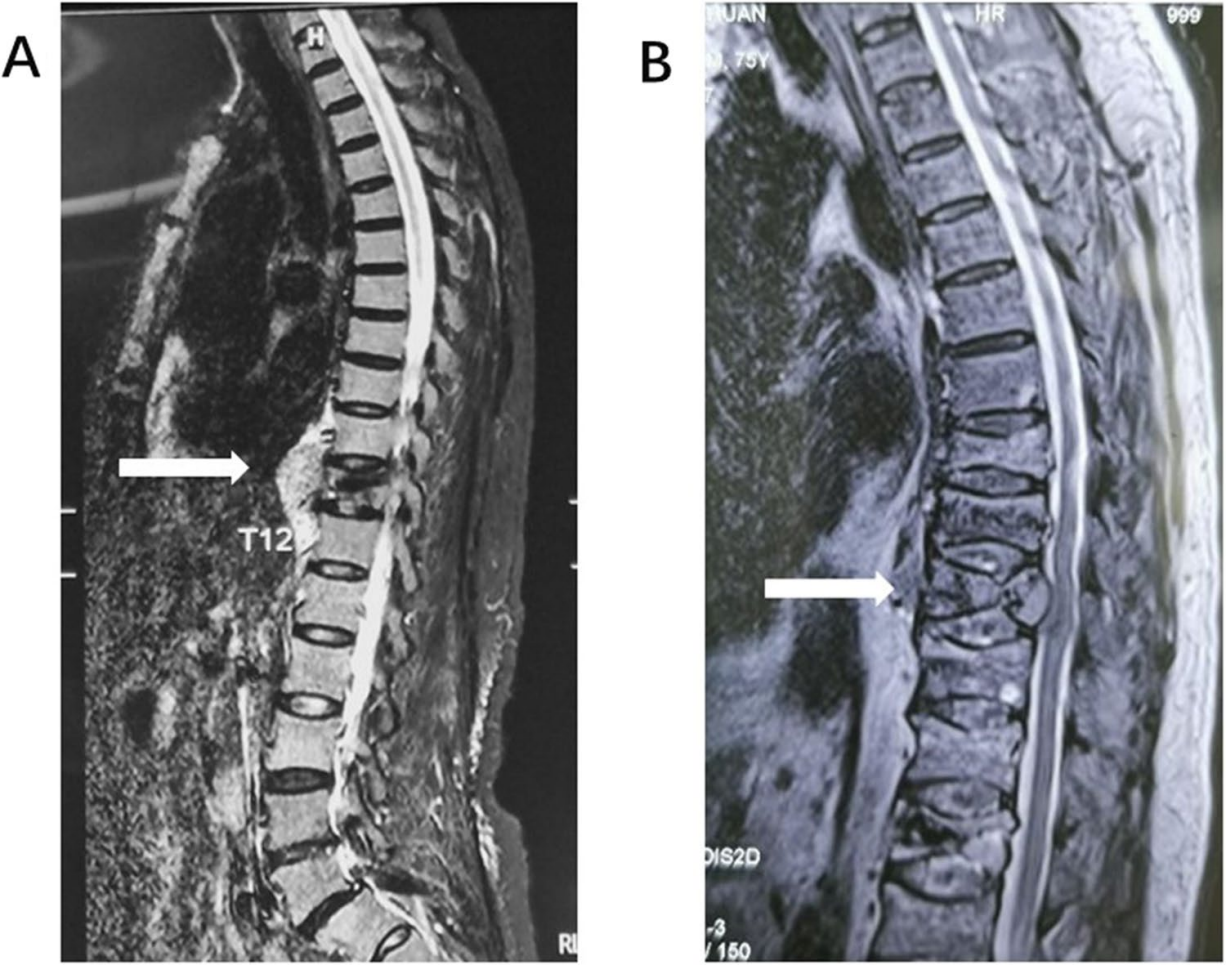
MRI images of case1 and case 2. **A** MRI image of case 1 shows tumor protrusion into the peritoneum. **B** MRI image of case 2 shows a T9 compression fracture with a soft tissue mass compressing the spinal cord

Mrs. A is a 69-year-old woman who presented to the orthopedic department on December 20, 2019, with back pain for 1 month, CT showed T11 vertebral fracture, and the patient’s back pain was relieved after PKP surgery. The patient was misdiagnosed with simple osteoporosis and underwent PKP surgery. The pain recurred and progressively worsened 3 months after surgery, and a thoracic spine magnetic resonance imaging (MRI) showed a mass in the T11 vertebral body area projecting into the retroperitoneum and partial spinal stenosis (Fig. 2). Subsequently, a puncture biopsy of the T11 vertebral lesion was performed, and the postoperative pathology showed plasmacytoid myeloma. The patient was admitted to the hematology department on April 29, 2020: blood routine—WBC 10.37 × 10*9/L, Hb 113 g/L, PLT 82 × 10*9/L, blood LAM 18.30 g/L, and light chain λ monoclonal bands were seen in both blood and urine immunofixation electrophoresis; bone marrow routine—plasma cells accounted for 73%; FISH (fluorescence in situ hybridization)—1q21 ≥ quadruple amplification. The patient was diagnosed with plasma cell leukemia (secondary) and multiple myeloma λ light chain type (DS stage IIIA, ISS stage II, and RISS stage II), after which the patient was treated with a regimen of izatzomib and liposomal adriamycin combined with dexamethasone. The patient had a progressive decrease in hemoglobin and platelets and developed a large amount of pleural fluid, and 6.93% clonal plasma cells were seen by flow cytometry immunotyping of the pleural fluid. The patient had a combination of pulmonary infection, respiratory failure, heart failure, rapid disease progression, and failed chemotherapy and died soon after, with an OS of 3.5 months.

#### Case 2

Mr. B is a 75-year-old man who presented to the orthopedic department on November 1, 2019, with a primary cause of low back pain for 1 month; CT showed T12 and L2 fracture, followed by PKP. There was pain relief after surgery. Seven months after surgery the patient developed progressive bilateral lower extremity numbness and weakness. MRI showed T9 vertebral compression fracture with soft tissue mass compressing the spinal cord (Fig. 2). PET-CT showed multiple osteolytic disruptions throughout the body. The patient was admitted to our department on August 7, 2020: bone marrow routine—plasma cells accounted for 12%. FISH did not detect MM-related chromosomal abnormalities; blood immunofixation electrophoresis—IgG-λ monoclonal bands were seen (β2 microglobulin 2.69 mg/L). There was the diagnosis of MM IgG-λ type (DS stage IIIA, ISS stage II, and RISS stage II). Ixazomib and liposomal adriamycin combined with dexamethasone regimen were given. Partial remission was achieved after 4 courses of treatment, but paraplegia did not improve, and the patient was continuously bedridden with a combined depressive state, refused further treatment, and died of pulmonary infection with an OS of 16.4 months.

### Prognosis

**Fig. 3:**
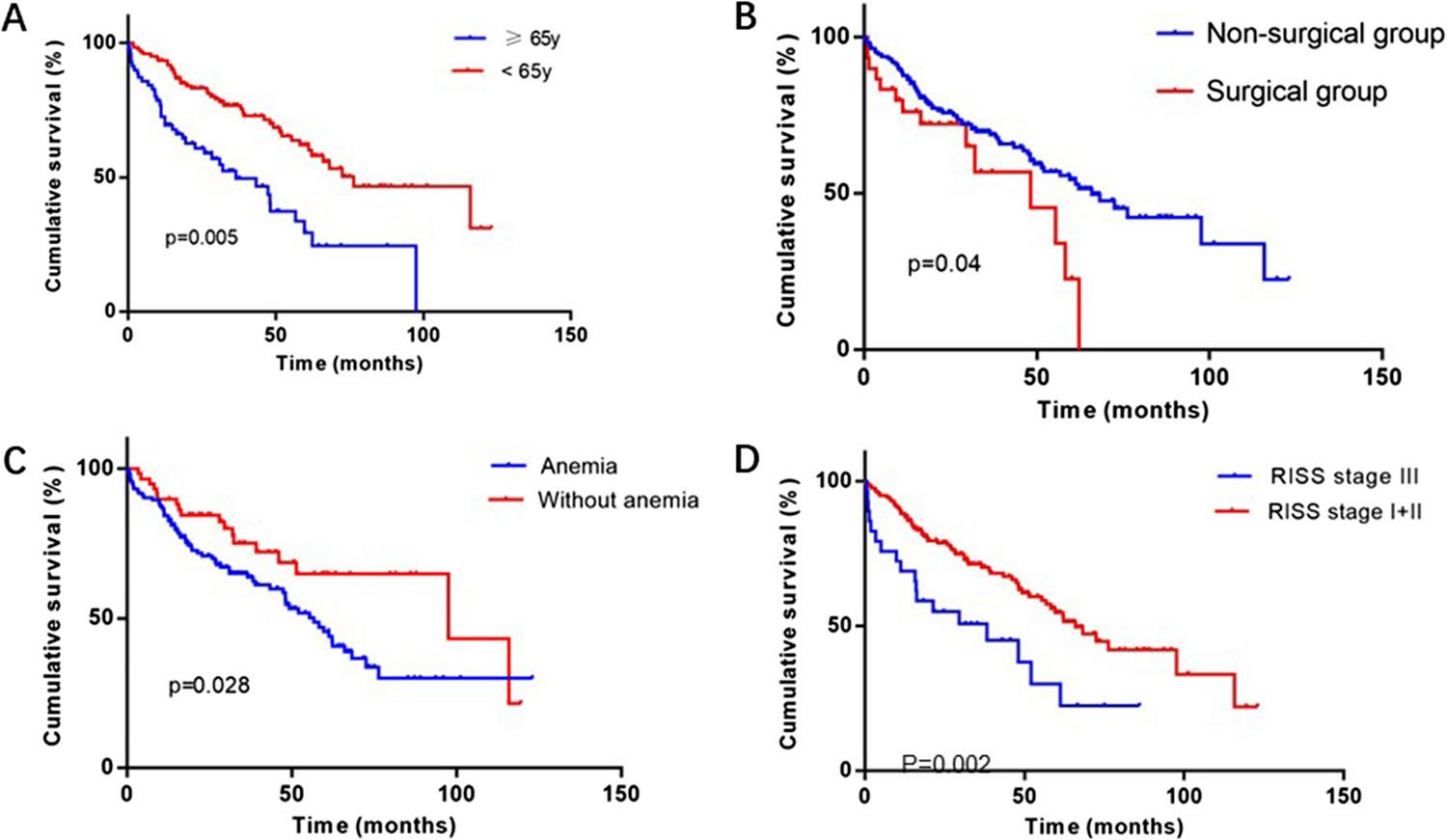
Kaplan–Meier curves of overall survival (OS). **A** Kaplan–Meier curves illustrating OS differences between age < 65 years and age ≥ 65 years. **B** Kaplan–Meier curves illustrating OS differences between surgical and non-surgical groups. **C** Kaplan–Meier curves illustrating OS differences between anemic and non-anemic patients. **D** Kaplan–Meier curves illustrating OS differences between RISS stage I + II patients and RISS stage III patients

The results of the survival analysis showed (Fig. 3) that the median follow-up was 32 months (0.3–123 months) and the median OS was significantly shorter in the surgical group than in the non-surgical group (48.2 months vs. 66 months, *p* = 0.04). Univariate analysis showed that surgery, age, anemia, and RISS stage were risk factors for OS, and further multifactorial analysis showed that age and RISS stage were independent prognostic factors for OS (Table 4).

**Table 4:** Analysis of risk factors for OS

	Univariate analysis		Multivariate analyses	
	HR	*p*	HR	*p*
Age	0.391 (0.251–0.608)	0.001	0.388 (0.24–0.628)	0.001
Gender	0.82 (0.531–1.269)	0.373		
Anemia	1.79 (1.058–3.03)	0.030	1.66 (0.958–2.877)	0.071
Renal insufficiency	0.981 (0.542–1.778)	0.951		
Hypercalcemia	1.35 (0.799–2.281)	0.262		
ISS staging	1.51 (0.978–2.33)	0.063		
RISS staging	2.242 (1.316–3.818)	0.003	2.669 (1.546–4.608)	0.001
Surgery	0.548 (0.305–0.983)	0.044	0.631 (0.343–1.161)	0.139

*ISS* International Staging System, *RISS* Revised International Staging System, *OS* overall survival

## Discussion

MBD seriously affects the quality of life. Vertebral fractures cause severe pain and limitation of motion, and patients desire immediate pain relief. Bisphosphonates, denosumab and antineoplastic drugs for MM can relieve bone pain, but the onset of action is slow. On this basis, combined PKP/PVP minimally invasive surgery is an effective treatment for rapid relief of bone pain in MM patients [22, 23] and also reduces the risk of complete bed rest and pulmonary infection, improves the quality of life of patients, and can lay the foundation for subsequent treatment [24]. A prospective randomized controlled trial enrolled 134 cancer patients (49 of whom were MM) and compared PKP surgery with nonsurgical treatment for painful vertebral compression fractures, finding that patients in the PKP surgery group achieved more rapid and sustained pain relief, significantly reduced time on pain medication and bed rest, and significantly improved quality of life compared to the nonsurgical group [25]. However, it should be noted that no surgical site biopsy was performed in this study, and the cause of vertebral fractures could not be determined to be caused by tumors, as radiation osteonecrosis, severe osteoporosis, or other causes of vertebral fractures in cancer patients can also occur in addition to cancer metastases. Several retrospective studies have also shown that PVP/PKP is safe and effective in MM patients, and it should be considered an effective treatment option for MM patients with vertebral compression fractures [9–16]. However, most of these studies were performed by orthopedic and radiologists, and there is a lack of hematologists to assess the disease state of MM, most MM patients received preoperative radiotherapy and chemotherapy, and the improvement in pain may not be entirely due to PVP/PKP surgery, as the treatment of MM disease and the use of bone-targeted drugs may reduce pain.

In this study, we reviewed the clinical data of patients with MM with vertebral fractures (*n* = 206) admitted to our center in the last decade. The results of the study showed that only less than 10% of patients in the surgical group obtained sustained pain relief after surgery, more than half of the patients obtained only short-term pain relief for less than 3 months, and about one-third of the patients had no pain relief after surgery, so the effect of surgical pain relief was not satisfactory. In patients with osteoporosis alone, the effect of PVP/PKP on pain relief has been inconsistently concluded in different studies, with most studies concluding that patients had significant pain relief within 1 year after surgery [26, 27]. However, some studies have also concluded that there was no significant improvement in pain in the surgical group compared to the placebo group [28]. A large sample study [29] in our spine surgery department analyzed the causes of low back pain in 1863 patients with osteoporotic vertebral compression fractures who still had pain after PKP/PVP. 1580 cases (84.8%) had pain relief after surgery, 283 cases (15.2%) had pain nonrelief, of which 32.2% (91/283) had pain nonrelief due to complications such as reoccurrence of fracture or infection after surgery relief, 2.1% (6/283) of the patients were misdiagnosed as osteoporotic vertebral compression fractures, 4 of the 6 cases eventually had a definite diagnosis of MM, and the other 2 cases were metastatic cancer. This shows that PVP/PKP for pain relief in patients with simple osteoporotic fractures is effective, but PVP/PKP for tumorigenic fractures without antitumor treatment is not effective, which is consistent with the finding of poor postoperative pain relief in patients in the surgical group in this study.

Thirty-two patients in the surgical group in this study were not treated preoperatively with anti-myeloma therapy because MM was not clearly diagnosed before PKP/PVP surgery. The incidence of new vertebral fractures after PKP/PVP in these patients was 74.2%, which was significantly higher than the probability of patients with osteoporosis alone (OVCF) (8.5%–29.8%) [30, 31]. Lee et al. [30] reported 402 patients with OVCF treated with PVP with a median follow-up of 4.8 years, during which 120 patients (29.8%) had recurrent vertebral fractures, 60% (72/120) of which were adjacent segmental fractures. Borensztein et al. [31] followed up 380 patients with OVCF treated with PVP and 30 (8.5%) had new vertebral fractures after 1 year. Buchbinder et al. [28] meta-analysis showed an 11% probability of new symptomatic vertebral fractures in OVCF patients 12–24 months after surgery (48/418). Only 5 (2.9%) of the 174 patients in the nonsurgical group in this study developed new vertebral fractures before receiving antimyeloma drugs, a significantly lower probability than in the surgical group (74.2%). In addition, the site of the new fracture had different characteristics between the two groups of patients. In the nonoperative group, 80% of patients had a new fracture site that was non-adjacent to the previous fracture site, whereas in the operative group, 66.7% of patients had a new vertebral fracture site that was adjacent to the previous fracture site. In a retrospective study [32], Sun et al. found that leakage of bone cement in the disc after PKP/PVP in patients with osteoporosis significantly increased the probability of fracture in the adjacent vertebral body. Previous studies have shown that cement injection increases the strength of the vertebral body but also increases the intervertebral pressure on adjacent vertebrae, changing the mechanics of the adjacent vertebrae and thus increasing the incidence of fractures in the adjacent vertebrae [33, 34]. A review of several studies suggests that it is difficult to assess the incidence of recurrent fractures after PKP/PVP in cancer patients, both because of the lack of follow-up data and because of the consideration that recurrent fractures may be the result of disease progression rather than a surgical complication[35]. In this study, both groups of patients were NDMM without antitumor treatment, and the incidence of new vertebral fractures was significantly higher in the surgical group than in the nonsurgical group considered to be related to surgical intervention, so that patients with a high risk of refracture after PKP/PVP surgery when the tumor was not controlled.

The IMWG consensus specifically emphasizes tumor control in MM patients before performing PKP/PVP surgery [17], but there are no data in the literature on the dangers of surgery in patients with uncontrolled myeloma. It has also been shown in some studies that early application of PVP/PKP in NDMM patients has no complications and does not affect subsequent myeloma treatment, but the small sample size and short follow-up period of this study do not provide sufficient evidence [36]. Our study adds to the data showing that patients with NDMM not treated with antimyeloma have limited pain relief with PVP/PKP surgery and a high risk of new vertebral fractures after surgery.

Two cases are specifically mentioned in this study; case 1 is characterized by the presence of 1q21 ≥ quadruple amplification and P53 deletion in the patient’s cytogenetics, defined as ultra-high-risk double-hit MM [37] with an extremely poor prognosis. This patient had rapid disease progression to plasma cell leukemia at the time of diagnosis of MM, and these clinical features were consistent with ultra-high cytogenetic risk. Bone cement filling occupies the tumor growth site, causing the tumor to grow convexly into the retroperitoneum. At the time of definitive diagnosis, the disease was in end stage, the patient’s general condition was poor, the tumor load was high, and the expected treatment outcome was poor. The surgery was done at a time when the diagnosis was not known. Although the surgery allowed the patient to have pain relief for 3 months, the diagnosis of MM was delayed and the best time for chemotherapy was missed. Case 2 is characterized by no significant cytogenetic abnormality in the patient, the biological malignancy is not high, and the prognosis is good if the myeloma is treated promptly. However, the patient did not seek further medical attention because of pain relief after surgery, after which the tumor progressed leading to paraplegia. Although subsequent chemotherapy brought the disease under control, the paraplegia could not be recovered and the quality of life was extremely poor, which seriously affected the patient’s willingness to follow up treatment and died of pneumonia caused by bed rest. Other studies have reported that MM patients misdiagnosed with simple osteoporosis underwent PVP/PKP surgery, resulting in incomplete paralysis of the lower extremities as the bone cement occupied the vertebral space and the tumor protruded into the spinal canal and grew [29]. Bone cement leakage is also a common complication after PVP/PKP, and the literature reports a 22.2% leakage rate of postoperative bone cement, with only 1.06% of those with symptoms [38]. In Ramos et al.’s study of PVP for MM, bone cement leakage rates of up to 84% were confirmed by postoperative CT [39]. No patients in this study experienced severe cement leakage, but data on asymptomatic minor cement leakage are not available due to the lack of orthopedic physician evaluation.

The median OS of the surgical group in this study was significantly shorter than that of the nonsurgical group. The results of multifactorial analysis showed that surgery was not an independent risk factor for OS in patients with MM, but surgery can mask the condition and delay the diagnosis. The results of the study showed that the time interval from the initial visit to the diagnosis of MM was significantly longer in the surgical group than in the nonsurgical group (6.1 months vs. 1 month, *p* < 0.01), and the delay in treatment led to an increased tumor load, which affected the outcome and quality of life of the patients. PVP/PKP is now widely used to treat vertebral compression fractures caused by osteoporosis. Osteoporosis is more prevalent in people over 55 years of age, and MM is equally prevalent in the middle-aged and elderly population. The results of a Chinese study [40] showed that 2.5% of patients (4/157) underwent PKP due to misdiagnosis of osteoporotic vertebral compression fracture, and the pathological findings by intraoperative biopsy showed 2 cases of MM, 1 case of Paget’s disease, and 1 case of chronic osteomyelitis. The results of foreign studies showed a 5.5% diagnosis rate of malignancy on routine biopsy during PKP [41]. Because routine intraoperative biopsy of PVP/PKP can safely detect unanticipated malignancies, preoperative evaluation and intraoperative biopsy should become routine to avoid misdiagnosis.

## Conclusion

The application of PKP/PVP surgery for pain relief in NDMM patients without antimyeloma therapy has a limited effect and a high risk of new vertebral fractures after surgery. Therefore, patients with NDMM may need to have their disease controlled with anti-myeloma therapy prior to any consideration for PKP/PVP surgery.

## Data Availability

The processed data required to reproduce these findings cannot be shared at this time as the data also is a part of an ongoing study.
